# Examining the health literacy and health behaviours of children aged 8–11 in Wales, UK

**DOI:** 10.1093/heapro/daaf026

**Published:** 2025-04-10

**Authors:** Emily Marchant, Emily Lowthian, Michaela James, Nia Davies, Tom Crick

**Affiliations:** Department of Education and Childhood Studies, Swansea University, Swansea, SA2 8PP, United Kingdom; Department of Education and Childhood Studies, Swansea University, Swansea, SA2 8PP, United Kingdom; National Centre for Population Health and Wellbeing Research, Swansea University Medical School, Swansea University, Swansea, SA2 8PP, United Kingdom; Faculty of Humanities and Social Sciences, Swansea University, Swansea, SA2 8PP, United Kingdom; Department of Education and Childhood Studies, Swansea University, Swansea, SA2 8PP, United Kingdom

**Keywords:** health literacy, health behaviours, schools, education, curriculum, curriculum for wales, health monitoring

## Abstract

Childhood is a period of significant formative development where knowledge, skills, and capacities for adequate health literacy are acquired, particularly within school settings. The new *Curriculum for Wales* (CfW), phasing in from September 2022 for learners aged 3–16 years, places statutory focus on health and well-being and school-level curriculum design, providing unprecedented opportunities to empower children as agents in making health-enhancing decisions. Designing, tracking, and evaluating impacts of the CfW on children’s health literacy requires scalable monitoring tools; however, research efforts have focused on adolescent populations. This national-scale scoping and pilot study, the first to explore children’s health literacy in Wales, piloted the *Health Literacy for School-Aged Children* (*HLSAC-5*) within the existing nationwide *Health and Attainment of Pupils in Primary EducatioN* (*HAPPEN-Wales*) health and well-being survey to examine the health literacy of children aged 8–11 (*n* = 2607) and explore associations between health literacy and health behaviours. Children’s health literacy was categorized as low (22.6%), moderate (50.4%), and high (27.0%). Multinomial logistic regression analyses suggest high health literacy compared to low health literacy was associated with higher sleep [relative risk ratio (RRR): 1.08, 95% CI 1.01–1.15], higher weekly physical activity (RRR: 1.13, 95% CI 1.03–1.25), fewer sedentary days per week (RRR: 0.89, 95% CI 0.81–0.99), and higher health-related well-being (RRR: 1.35, 95% CI 1.27–1.44). This study offers a sustainable measure of pre-adolescent children’s health literacy and health behaviours and tracking of CfW impacts. This enables efforts to be tailored to *person-centred* (understanding children’s health literacy needs), *place-based* (examining specific organizational health literacy context within schools and CfW design), and *policy-focused* approaches (re-energizing health literacy within current/emerging policies in Wales including the CfW).

**CONTRIBUTION TO HEALTH PROMOTION STATEMENT**

- First study of its kind that captures and explores children’s health literacy needs in Wales, UK.
- Fills a gap in understanding of health literacy below adolescent age.
- Offers a scalable and sustainable measure of pre-adolescent children’s health literacy, contributing to knowledge across three themes:
  - ◦ *Person-centred*: understanding the specific health literacy and health behaviour challenges of children in Wales.
  - ◦ *Place-based*: identifying key contextual health behaviours to better inform school-level health literacy curriculum design.
  - ◦ *Policy-focused*: enabling impact tracking of emerging national-level policy (e.g. the new *Curriculum for Wales*) with statutory health and well-being focus and potential of health literacy.

## BACKGROUND

Health literacy is a modifiable factor that contributes to the promotion and maintenance of good health and well-being throughout an individual’s life. The World Health Organization (WHO) identified health literacy as a global priority and advocates that it is a fundamental competency necessary to function within modern society (WHO 2023a). As such, health literacy has become prominent within a range of international policy and strategy (Marchant and Crick 2023, Marchant 2024). In Wales, UK, it is recognized as ‘the ability and motivation level of an individual to access, understand, communicate and evaluate both narrative and numeric information to promote, manage and improve their health status throughout their lifetime’ (Puntoni 2010). A growing body of evidence demonstrates the importance of health literacy on a range of health outcomes and healthcare savings costs (Shahid *et al*. 2022). Those with low health literacy struggle to understand and use information to make decisions about their health, engage in fewer health-promoting behaviours, and report lower health status (Von Wagner *et al*. 2007). Health literacy is unequally distributed across populations, mirroring the social gradient of wider health outcomes and health behaviour research (Sørensen *et al*. 2015). Thus, one of the greatest potential outcomes of improving population health literacy is reducing health inequities and inequalities.

In addressing population health literacy, it is important to consider the diverse and varying health literacy needs specific to contexts and target groups (WHO Regional Office for Europe 2019). At the individual level, health literacy is viewed as a personal asset that is developed and enhanced throughout the life course (Clouston *et al*. 2017). While attention to date has been placed heavily on adult populations, with evidence suggesting around 4 in 10 adults have low health literacy (Baccolini *et al*. 2021), research on children’s health literacy has gained momentum more recently. These efforts have been weighted more towards adolescent age (Bröder *et al*. 2017, Guo *et al*. 2018, Okan *et al*. 2018); data from 10 European countries suggests that up to 17.7% of adolescents have low health literacy (Paakkari *et al*. 2020). However, there is a significant gap in understanding the health literacy needs and associated health behaviours below adolescent age, particularly in those aged 12 and below (Guo *et al*. 2018, Okan *et al*. 2018). This is in part due to a lack of validated measurement tools in capturing and monitoring health literacy within this age group (Bánfai-Csonka *et al*. 2022, Marchant and Crick 2024). Childhood is a period of significant formative development where knowledge, skills, and capacities instrumental for adequate health literacy are acquired. A number of developmental stages occur during childhood, with middle childhood as a transitional pre-adolescent period between the ages of 6 and 12 years. During middle childhood, children are becoming increasingly responsible for their own health (Velardo and Drummond 2017) and are establishing health behaviours that impact their health and well-being throughout the life course (Bröder *et al*. 2017).

Academics have advocated for research to extend beyond solely judging levels of health literacy, towards taking a child-centred approach that consider children’s own views, actions, and wider behaviours associated with health literacy levels (Velardo and Drummond 2017). The Organisation for Economic Co-operation and Development (OECD) have developed a framework for measuring what matters most to children to support research efforts that aim to advance understanding and develop better policies to promote children’s health (OECD 2021). This framework advocates for more age-specific and child-reported measures that employ a multidimensional approach by considering the multiple indicators spanning health behaviours, perceptions, and well-being (OECD 2021). In doing so, health literacy can be supported and enhanced at different developmental stages tailored to need and context, in addition to informing the design and implementation of policy and practice. During the pre-adolescent developmental stage, schools are one of the key settings where children obtain health information from, for wider health promotion and health literacy activities to be delivered, and for monitoring approaches to be targeted (Brown *et al*. 2006, Van Boxtel *et al*. 2024).

Schools and curricula offer opportunities to develop the skills needed for accessing, understanding, appraising, and applying health information (Paakkari and Paakkari 2012, Marchant and Crick 2024, Van Boxtel *et al*. 2024). Health literacy is viewed as both a learning process and outcome (Paakkari and Paakkari 2012, Van Boxtel *et al*. 2024), and has been embedded within education policies and systems globally during periods of curriculum reform. This includes countries such as Finland and Australia where health literacy is a key learning outcome, curriculum component, and overarching theoretical framework (Marchant and Crick 2024). In Wales, progress in the field of health literacy has stalled since identified as a priority in 2010 (Puntoni 2010). However, there has been a more recent broader policy shift to reflect health, well-being, and education as fundamental national priorities (Welsh Government 2015, 2019a, 2021, 2023a, Marchant 2024), matching developments in international research, policy, and strategy (WHO Regional Office for Europe 2019, Council of Europe 2023). While this is a positive shift in policy, the importance of health literacy as a tool for health promotion and advocacy cannot be underscored. This is particularly important in the context of health, well-being, and education outcomes of children in Wales; low achievement results through the Program for International Student Assessment (PISA) have been observed since 2006 (Senedd Research 2023), a quarter of children are overweight or obese (Public Health Wales NHS Trust 2023), and almost half of children (49%) do not meet physical activity guidelines (Richards *et al*. 2022). Thus, enhancing health literacy as a vehicle to address these national priorities must be considered at the individual, organizational, and wider policy level, including developing health literacy as a personal asset and ensuring places and policies are conducive to enhancing health literacy (Rowlands *et al*. 2018, Dadaczynski *et al*. 2022). This is relevant in terms of developing *person-centred* health literacy at the individual child level, *place-based* health literacy through organizational school health literacy approaches such as curriculum design, and *policy-focused* health literacy through policy commitments and the potential of health literacy as a key driver of achieving national health, well-being, education, and economic policy visions (Sørensen *et al*. 2021, Marchant 2024).

The most promising avenue in Wales for strengthening the health literacy of current and future generations through *person-centred, place-based*, and *policy-focused* approaches is through ongoing major education system-level reforms and the implementation of the new *Curriculum for Wales* (CfW) (Welsh Government 2020). Rolled out from September 2022 onwards for learners aged 3–16 years as part of a ‘national mission’ (Welsh Government 2023b, Crick *et al*. 2024), the CfW places renewed statutory focus on health and well-being and offers autonomy to schools for school-level curriculum design tailored to need (Welsh Government 2022). Within the CfW, developing *healthy, confident individuals* is one of *Four Purposes* overarching the curriculum, and *Health and Well-being* constitutes one of six *Areas of Learning and Experience* (*AoLE*) through which teaching and learning is delivered (Welsh Government 2020, Lewis and Crick 2022). This aims to provide all school-aged children with the knowledge, skills, and competencies to empower them as agents in making health-enhancing decisions impacting themselves and others. This school-level autonomy intends to enable schools to tailor curriculum design to their children’s specific needs, thus understanding health literacy in this context are fundamental to a tailored *Health and Well-being AoLE*. Progression through the *Health and Well-being AoLE* mirrors a continuum of health literacy development, with alignment to Nutbeam’s typology of functional, interactive, and critical domains (Nutbeam 2000), and Paakkari and Paakkari’s theoretical framework of health literacy as a learning outcome (Paakkari and Paakkari 2012). Thus, the new CfW offers significant opportunities to develop and enhance health literacy, embedding health literacy as a learning process and measurable outcome of education in Wales (Nutbeam 2009). Meaningfully designing a school-level curriculum tailored to children’s health, well-being, and health literacy needs, in addition to evaluating these impacts, requires scalable and sustainable data collection and infrastructure.

In the 2010 health literacy scoping review in Wales, the authors called for the development of a national assessment method to provide a greater understanding of population health literacy and monitor the impacts of any health literacy-specific interventions (Puntoni 2010). The CfW and its renewed statutory focus on health and well-being could be viewed as such an intervention, and examining these impacts requires the implementation of health literacy monitoring tools. Indeed, the development of brief assessment tools that capture health literacy as both a learning process and an outcome and are integrated within longer surveys has been observed elsewhere (Paakkari *et al*. 2016, 2023, Marchant and Crick 2024). In the context of Wales, the use of scalable, health-related, self-reported surveys administered in schools are demonstrated with high uptake. This includes the *Health and Attainment of Pupils in Primary EducatioN* (*HAPPEN-Wales*) platform, a research and education infrastructure which currently engages with over half of all 1200 primary schools in Wales (Marchant 2020, HAPPEN Wales 2023, Einhorn *et al*. 2024, Locke *et al.* 2024). However, there is currently no assessment of health literacy of children and young people underway in Wales, limiting our understanding of children’s health literacy needs and schools’ ability to meaningfully design learner-tailored health and well-being curriculum components. Furthermore, this inhibits opportunities to track policy implementation and specific CfW impacts on children (Marchant and Crick 2024). Capitalizing on this by incorporating brief measures of health literacy within existing, established nationwide surveys is a feasible avenue to explore, and, given the broader policy shift in this direction, can position Wales internationally as a national policy testbed case study (Marchant 2024). This can be used to inform the tailored design and evaluation of health literacy interventions at local, regional, and national level, identify policymaking priorities and in the context of Wales, and examine the impact of statutory health and well-being curriculum focus on children’s health literacy.

The significance of this study is positioned within developing a greater understanding and tracking of health literacy need within middle childhood, given the lack of research focused to this age group. During middle childhood, children are gaining increasing independence and agency as they transition to adolescence. This is a period of formative development where knowledge, skills, and capacities crucial for optimal health literacy are developed, and health behaviours are established that can be tracked into adolescence and adulthood that influence health and well-being throughout the life course (Bröder *et al*. 2017).

This study, the first to explore health literacy among children in Wales, aimed to pilot the integration of a measure of health literacy in school-aged children within an existing nationwide health and well-being survey for primary school children in Wales in order to:

(i) Examine the health literacy of primary school-aged children (aged 8–11);
(ii) Explore associations between health literacy and health behaviours.

## METHODS

### Study design

This pilot study was conducted through the *HAPPEN-Wales* platform. *HAPPEN-Wales* was established in Wales, UK in 2014 following research with headteachers who advocated for increased collaboration to prioritize pupils’ health and well-being (Christian *et al*. 2015, Todd *et al*. 2015, 2016) and runs up to the current date. *HAPPEN-Wales* is a platform for conducting school-based research (Marchant *et al*. 2019, 2020, 2021, 2022a, 2022b, 2024, James *et al*. 2021a, 2021b, 2022, Einhorn *et al*. 2024); since the start of the implementation of the new CfW in 2022, it aims to support schools with *Health and Well-being Area of Learning and Experience* curriculum design tailored to children’s need. Through *HAPPEN-Wales*, school children aged 8–11 years (school years 4–6) complete the *HAPPEN* survey, an online survey that captures a range of validated self-reported health behaviours including physical activity, nutrition, and sleep (Everson *et al*. 2019). School participation in *HAPPEN-Wales* is voluntary and is either once, annually or biannually (e.g. to evaluate school-based interventions). Schools in this pilot study were recruited in April 2023 through *HAPPEN-Wales.* Participants completed the *HAPPEN survey* with the integration of a measure of health literacy between 4 May and 14 July 2023 or 16 April and 12 July 2024.

### The *HAPPEN* Survey and *Health Literacy for School-Aged Children* instrument

The *HAPPEN* survey is an online self-report survey that captures a range of typical health behaviours and well-being indicators. These include measures of weekly physical activity, sedentary and sleep behaviours, fruit, vegetable, carbonated drink and confectionary (sugary snack) consumption, dental health, autonomy, general and physical competency, and well-being (Everson *et al*. 2019). The survey is conducted online and completed by the child during school time. The *HAPPEN* survey promotes knowledge exchange and tailored curriculum design through a data collection and feedback system. Group-level health and well-being data obtained through the *HAPPEN* survey is shared with schools as a school report to inform local CfW design and implementation (Welsh Government 2020). Annual whole sample reports are also shared with key stakeholders in health and education for regional and national planning. A full copy of the survey is presented in Supplementary File A.

For this pilot study, a literature review of validated tools assessing school-aged children’s health literacy was undertaken (conducted by N.D. and E.M.) to identify a measure for integration within the existing *HAPPEN* survey. This process identified the *Health Literacy for School-Aged Children* (*HLSAC*), originally a 10-item instrument developed in Finland by Paakkari and colleagues (Paakkari *et al*. 2016). The *HLSAC* is based on the conceptualization of health literacy as a learning outcome in schools across five core domains: *theoretical knowledge*, *practical knowledge*, *critical thinking*, *self-awareness*, and *citizenship* (Paakkari and Paakkari 2012). This conceptualization and its competencies strongly align with the new CfW philosophy and approach. This brief measure has been included within other existing population-level health-related surveys including the international WHO-endorsed *Health Behaviour in School-aged Children* (*HBSC*) survey (WHO 2024). Given the need for this pilot study to identify a brief school and curriculum context-specific measure of children’s health literacy suitable for integration within an existing survey, the *HLSAC* was deemed the most suitable tool to be embedded within the *HAPPEN* survey.

The original *HLSAC* consists of 10 items, with each domain of *theoretical knowledge*, *practical knowledge*, *critical thinking*, *self-awareness*, and *citizenship* represented by two items. Validation analyses of the 10-item *HLSAC* demonstrates high internal consistency (high Cronbach’s α: 0.93) (Paakkari *et al*. 2016). The tool has been translated and piloted in a number of European countries (including Norway, Finland, Poland, Slovakia, Germany, Estonia, Czechia, Belgium) and therefore provides opportunities for pan-Europe comparisons (Paakkari *et al*. 2018, 2019b, Bjørnsen *et al*. 2022, Bonde *et al*. 2022, Fischer *et al*. 2022). More recently, the 10-item *HLSAC* has been refined, and a brief five-item *HLSAC* measure, using one item for each of the health literacy conceptual domains, has been developed (Paakkari *et al*. 2023). Using data from seven European countries, confirmatory factor analysis confirms structural validity with a good model fit across countries and the total sample (comparative fit index = 0.995, Tucker Lewis Index = 0.989, standardized root mean square residual = 0.011, root mean square error of approximation = 0.031), and high internal consistency (α = 0.87–0.98) across countries (Paakkari *et al*. 2023). For the *HLSAC-5*, sum scores are calculated, and health literacy is classified as low (score: 5–12), moderate (score: 13–17), or high (score 18–20). Validation research shows good structural and criterion validity and good internal consistency (Paakkari *et al*. 2023). The *HLSAC-5* provides new opportunities to be integrated into existing longer surveys, enabling the simultaneous measurement of multiple health-related concepts including health literacy, health behaviours, and well-being (Paakkari *et al*. 2023). This addresses previous limitations impacting survey reliability and response rate observed with the administration of multiple surveys capturing different health-related measures (Porter *et al*. 2004).

Before integration within the *HAPPEN* survey, minor amendments to wording were made to three of five items within the *HLSAC-5* to improve contextual readability, specifically relating to English language. Minor conceptual adjustments to wording have been observed in the translation process of the *HLSAC-5* in other countries (Bjørnsen *et al*. 2022). For example, in the current study, the original item ‘I have good information about my health’ was re-worded in the *HAPPEN* survey as ‘I have good knowledge about my health’. Minor amendments were made by the lead researcher (E.M.) and confirmed with members of the project team (M.J. and T.C.). A copy of the 10-item and 5-item *HLSAC* is presented in Supplementary File B, including both the original and current study versions. For the purpose of this study and for future data collection, analyses, and comparison, the *HLSAC-5* scores were considered as the primary outcome.

### Participants

Recruitment for this pilot study was achieved through *HAPPEN-Wales* via a number of methods including e-mail, social media promotion, and through stakeholders in health and education (including local government health and well-being teams, as well as regional education consortia). This contact included information about the pilot study, and interested primary schools were then invited to share details of the *HAPPEN* survey (including study aims and a parent information sheet) with parents/guardians and the opportunity to opt their child out from the survey. Child consent was also obtained at the start of the survey. In total, 2607 school-aged children from 50 primary schools completed the *HAPPEN* survey with complete *HLSAC-5* data. In accordance with the *HAPPEN* model and for the purpose of knowledge of exchange and CfW design, participating schools received individualized school reports presenting group-level data of their pupils’ health and well-being indicators with the addition of health literacy information obtained through the *HLSAC-5*.

### Ethics

Ethical approval was granted by the Swansea University Medical School Research Ethics Committee (2017-0033J, 18 April 2023). All participants were assigned a unique anonymizing ID number, and any personal data such as names were removed. Electronic data (survey responses) were stored in secure, password-protected files that were only accessible to the research team.

### Quantitative analysis

The process of data coding involved two researchers. The first researcher (M.J.) downloaded the raw data, cleaned the data, checked for duplicates, generated a unique participant ID number, and removed identifiable information to protect participants’ anonymity. The raw dataset was coded by the second researcher (E.M.) for the purpose of data analyses.

Data were managed and analysed on SPSS 29 (IBM Corp. 2023) and R (R Core Team 2021). Multinomial logistic regression analyses examined relative risk ratios (RRRs) of the primary outcome using the ‘nnet’ package (Venebles and Ripley 2002); participants’ levels of health literacy obtained from the *HLSAC-5* categorized as ‘low’, ‘moderate’, or ‘high’; we had a Cronbach alpha of 0.88 suggesting high internal consistency. The selection of independent variables from the *HAPPEN* survey were theoretically informed based on indicators recognized by the OECD as typical childhood health behaviour and well-being domains that warrant research (OECD 2021). These variables included nutrition (daily fruit and vegetable consumption, weekly confectionary snack consumption, and carbonated drink consumption), weekly physical activity (≥ 60 min), sedentary behaviours (≥ 120 min), sleep hours, ability to ride a bike, and health-related well-being. Analyses were adjusted for confounding variables (sex, academic school year, area-level deprivation using the Welsh Index of Multiple Deprivation [WIMD]) (Welsh Government 2019b).

Due to the proportion of missing data in some of the covariates (0.4%–51.2%, see Table 1), missing data were multiply imputed using R’s *mice* and *miceadds* (van Buuren and Groothuis-Oudshoorn 2011) package using chained equations. We adjusted for the different variable types (binary, categorical, continuous), involved all variables in the model, and computed 10 imputations with 10 iterations per imputation. Complete-case models and imputed models were compared to explore any differences in estimates; due to bias likely introduced in complete-case models, imputed models are shown in the results with complete-case models in Supplementary File C.

**Table 1.** Descriptive statistics of the study sample by whole sample and by low, moderate, and high health literacy group using the *HLSAC-5* [data are presented as *n* (%) and mean ± SD]

	Whole sample	Low health literacy	Moderate health literacy	High health literacy
Participants	2607	590 (22.6%)	1314 (50.4%)	703 (27.0%)
Gender				
Female	1246 (48.2%)	253 (43.2%)	649 (49.8%)	344 (48.2%)
Male	1215 (47.0%)	299 (51.0%)	588 (45.2%)	328 (47.0%)
Prefer not to say	125 (4.8%)	34 (5.8%)	65 (5.0%)	26 (4.8%)
Missing	21 (0.8%)			
School year				
Year 3	144 (5.5%)	42 (7.2%)	60 (4.6%)	42 (6.0%)
Year 4	746 (28.7%)	159 (27.2%)	371 (28.3%)	216 (30.8%)
Year 5	785 (30.2%)	187 (32.0%)	392 (29.9%)	206 (29.4%)
Year 6	922 (35.5%)	197 (33.7%)	488 (37.2%)	237 (33.8%)
Missing	10 (0.4%)			
WIMD quintile				
1 (most deprived)	246 (19.3%)	50 (18.9%)	138 (20.9%)	58 (16.7%)
2	202 (15.9%)	54 (20.4%)	110 (16.7%)	38 (10.9%)
3	294 (23.1%)	58 (21.9%)	149 (22.6%)	87 (25.0%)
4	305 (24.0%)	68 (25.7%)	169 (25.6%)	68 (19.5%)
5 (least deprived)	226 (17.8%)	35 (13.2%)	94 (14.2%)	97 (27.9%)
Missing	1334 (51.2%)			
Ethnicity				
Asian	85 (3.3%)	22 (3.8%)	46 (3.5%)	17 (2.4%)
Black	121 (4.7%)	28 (4.8%)	51 (3.9%)	42 (6.0%)
White	1958 (75.6%)	427 (73.2%)	991 (75.9%)	540 (77.1%)
Mixed	177 (6.8%)	41 (7.0%)	97 (7.4%)	39 (5.6%)
Prefer not to say	248 (9.6%)	65 (11.1%)	121 (9.3%)	62 (8.9%)
Missing	18 (0.7%)			
Daily fruit and vegetable consumption	3.12 ± 2.03	3.05 ± 2.21	2.99 ± 1.92	3.44 ± 2.07
Missing	55 (2.1%)			
Daily toothbrushing				
0	95 (3.7%)	38 (40.0%)		14 (14.7%)
1	516 (20.1%)	135 (26.2%)	43 (45.3%)	108 (20.9%)
2	1781 (69.5%)	360 (20.2%)	273 (52.9%)	521 (29.3%)
3	171 (6.7%)	47 (27.5%)	900 (50.5%)	49 (28.7%)
Missing	44 (1.7%)		75 (43.9%)	
Sleep hours	8.8 ± 2.08	8.4 ± 2.35	8.76 ± 2.06	9.19 ± 1.82
Missing	55 (2.1%)			
Weekly physical activity (≥ 60 min)				
0 days	179 (6.9%)	56 (9.6%)	97 (7.4%)	26 (3.7%)
1–2 days	656 (25.4%)	173 (29.6%)	336 (25.8%)	147 (21.1%)
3–4 days	662 (25.6%)	109 (18.6%)	389 (29.9%)	164 (23.5%)
5–6 days	468 (18.1%)	97 (16.6%)	224 (17.7%)	147 (21.1%)
7 days	620 (24.0%)	150 (25.6%)	257 (19.7%)	213 (30.6%)
Missing	22 (0.8%)			
Weekly sedentary/screen time (≥ 120 min)				
0 days	128 (5.0%)	21 (3.6%)	62 (4.8%)	45 (6.5%)
1–2 days	480 (18.7%)	96 (16.7%)	238 (18.4%)	146 (21.0%)
3–4 days	525 (20.5%)	119 (20.7%)	260 (20.1%)	146 (21.0%)
5–6 days	374 (14.6%)	73 (12.7%)	198 (15.3%)	103 (14.8%)
7 days	1055 (41.2%)	267 (46.4%)	534 (41.3%)	254 (36.6%)
Missing	45 (1.7%)			
Weekly carbonated drink consumption (≥ 1 per day)				
0 days	827 (32.3%)	185 (32.0%)	407 (31.4%)	235 (34.1%)
1–2 days	1015 (39.6%)	209 (36.1%)	518 (40.0%)	288 (41.8%)
3–4 days	396 (15.4%)	88 (15.2%)	221 (17.7%)	87 (12.6%)
5–6 days	127 (5.0%)	26 (4.5%)	68 (5.2%)	33 (4.8%)
7 days	199 (7.7%)	71 (12.3%)	82 (6.3%)	46 (6.7%)
Missing	43 (1.6%)			
Weekly confectionary snack consumption (≥ 1 per day)				
0 days	188 (7.3%)	52 (9.1%)	87 (6.7%)	49 (7.1%)
1–2 days	775 (30.3%)	158 (27.5%)	390 (30.1%)	227 (32.9%)
3–4 days	699 (27.3%)	139 (24.2%)	391 (30.2%)	169 (24.5%)
5–6 days	358 (14.0%)	89 (15.5%)	173 (13.3%)	96 (13.9%)
7 days	539 (21.1%)	136 (23.7%)	255 (19.7%)	148 (21.5%)
Missing	48 (1.8%)			
Ride a bike				
Yes	2078 (83.9%)	459 (81.1%)	1034 (83.2%)	585 (87.6%)
No	399 (16.1%)	107 (18.9%)	209 (16.8%)	83 (12.4%)
Missing	130 (5.0%)			
Health-related well-being	7.93 ± 2.32	7.27 ± 2.80	7.75 ± 2.21	8.80 ± 1.78
Missing	51 (2.0%)			

## RESULTS

Table 1 presents the descriptive statistics of the study sample by whole sample and by low, moderate, and high health literacy group using the *HLSAC-5*. Survey responses were obtained from 2607 participants (48.2% girls, *n* = 1246; 47.0% boys, *n* = 1215; 4.8% prefer not to say, *n* = 125). Within the sample, 22.6% had low health literacy (*n* = 590), 50.4% had moderate health literacy (*n* = 1314), and 27.0% high health literacy (*n* = 703).

**Figure 1.**
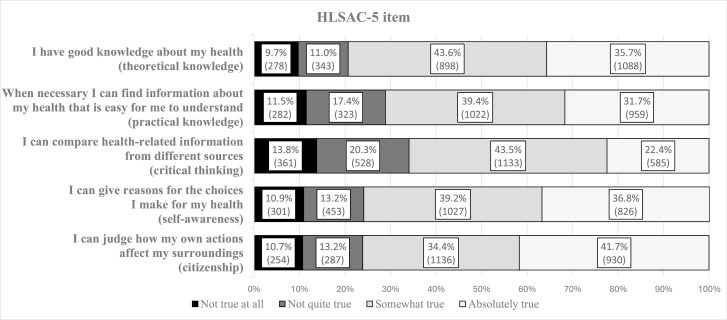
*HLSAC-5* matrix items, *n* (%).

Figure 1 presents a matrix of *HLSAC-5* survey item responses from the sample of 2607 children. Of the children, 20.7% responded to the statement *I have good knowledge about my health* as *not true at all/not quite true*, representing *theoretical knowledge* and more functional domains of health literacy (Nutbeam 2000). For practical knowledge, 28.9% reported *not true at all/not quite true* for the statement *when necessary, I can find information about my health that is easy for me to understand.* Regarding critical thinking, 34.1% responded *not true at all/not quite true* regarding their ability to *compare health-related information from different sources*, indicating lower levels of critical health literacy according to Nutbeam’s conceptualization (Nutbeam 2000). For the *self-awareness* and *citizenship* theoretical survey components, 24.1% and 23.9% reported *not true at all/not quite true* to being able to *give reasons for the choices I make* and *I can judge how my own actions affect my surroundings*, respectively.

Table 2 presents the imputed multinomial logistic regression for comparisons against low health literacy (base outcome), moderate health literacy, and high health literacy using the *HLSAC-5* with health behaviours and health-related well-being; we present coefficients as RRRs. For those categorized as having moderate health literacy compared to low, there was a statistically significant lower risk of being male (RRR: 0.79, *P* < 0.05, 95% CI 0.64–0.99), and significantly higher risks of brushing their teeth twice daily compared to zero (RRR: 1.72, *P* < 0.05, 95% CI 1.04–2.84) and higher health-related well-being (RRR: 1.08, *P* < 0.01, 95% CI 1.03–1.12). Those categorized as having high health literacy compared to low were significantly less likely to be male (RRR: 0.77, *P* < 0.05, 95% CI 0.60–1.00) and more likely to be in the least deprived WIMD quintile (quintile 5) compared to the most deprived (quintile 1) (RRR: 1.90, *P* < 0.05, 95% CI 1.06–3.41). Regarding health behaviours, high health literacy had significant associations with having more sleep (RRR: 1.08, *P* < 0.05, 95% CI 1.01–1.15), higher weekly physical activity (RRR: 1.13, *P* < 0.01, 95% CI 1.03–1.25), fewer sedentary days per week (RRR: 0.89, *P* < 0.05, 95% CI 0.81–0.99), and higher health-related well-being (RRR: 1.35, *P* < 0.01, 95% CI 1.27–1.44). The majority of our coefficients were within +0.30/−0.31; however, some coefficients had a larger difference including tooth brushing for the high models (+1.96/−8.04), and ethnicity in both models (−0.55/−0.78). It is important to note that to date, the tool has been validated for use in childhood populations aged 11 and above, and this is the first study to use the *HLSAC-5* in this population.

**Table 2.** Multinomial logistic regression model of *HLSAC-5* low (base outcome), moderate, and high health literacy with typical childhood health behaviour trends, well-being, and perception-related domains

Reference: low health literacy	Estimate	SE	*P*	95% CI	
				Lower bound	Upper bound
Moderate health literacy					
School year	1.11	0.06	0.07	0.99	1.24
Male	0.79	0.11	0.04*	0.64	0.99
Reference: female	1.00				
Ethnicity					
Black	0.87	0.36	0.69	0.43	1.76
White	1.16	0.25	0.57	0.70	1.90
Mixed	1.22	0.30	0.51	0.68	2.22
Reference: Asian	1.00				
WIMD quintile					
2	0.71	0.21	0.11	0.47	1.08
3	0.90	0.20	0.61	0.61	1.34
4	0.82	0.21	0.36	0.53	1.27
5 (least deprived)	0.92	0.24	0.74	0.57	1.49
Reference: 1 (most deprived)	1.00				
Fruit and vegetable consumption	0.98	0.03	0.36	0.92	1.03
Daily toothbrushing frequency					
1	1.57	0.26	0.09	0.94	2.62
2	1.72	0.26	0.04*	1.04	2.84
≥ 3	1.15	0.32	0.66	0.62	2.16
Reference: 0	1.00				
Hours of sleep	1.03	0.03	0.29	0.98	1.08
Days physically active in previous 7 days	0.97	0.05	0.51	0.89	1.06
Reference: 0 days	1.00				
Days sedentary in previous 7 days	0.95	0.04	0.23	0.87	1.03
Reference: 0 days	1.00				
Days consumed carbonated drink in previous 7 days	0.93	0.04	0.12	0.86	1.02
Reference: 0 days	1.00				
Days consumed confectionary snack in previous 7 days	0.96	0.05	0.35	0.88	1.05
Reference: 0 days	1.00				
Ride a bike	1.09	0.14	0.56	0.82	1.44
Reference: Can’t ride a bike	1.00				
Health-related well-being	1.08	0.02	0.00*	1.03	1.12

High health literacy					
School year	1.08	0.07	0.25	0.95	1.24
Male	0.77	0.13	0.05*	0.60	1.0
Reference: female	1.00				
Ethnicity					
Black	2.15	0.43	0.08	0.92	5.04
White	1.78	0.35	0.10	0.90	3.501
Mixed	1.20	0.40	0.64	0.55	2.63
Reference: Asian	1.00				
WIMD quintile					
2	0.59	0.26	0.04	0.35	0.99
3	1.22	0.24	0.42	0.75	1.96
4	0.70	0.29	0.23	0.38	1.28
5 (least deprived)	1.90	0.29	0.03*	1.06	3.41
Reference: 1 (most deprived)	1.00				
Fruit and vegetable consumption	1.02	0.03	0.49	0.96	1.09
Daily toothbrushing frequency					
1	1.47	0.36	0.28	0.73	2.98
2	1.79	0.34	0.09	0.91	3.52
≥ 3	1.13	0.40	0.76	0.51	2.48
Reference: 0	1.00				
Hours of sleep	1.08	0.03	0.02*	1.01	1.15
Weekly days physically active	1.13	0.05	0.01*	1.03	1.25
Reference: 0 days	1.00				
Sedentary days per week	0.89	0.05	0.03*	0.81	0.99
Reference: 0 days	1.00				
Weekly days consumed carbonated drink	0.94	0.06	0.26	0.84	1.05
Reference: 0 days	1.00				
Weekly days consumed confectionary snack	0.99	0.06	0.79	0.88	1.10
Reference: 0 days	1.00				
Ride a bike	1.31	0.18	0.13	0.92	1.86
Health-related well-being	1.35	0.03	0.00*	1.27	1.44

*P < 0.05.

## DISCUSSION

This is of the first study of its kind to capture the health literacy needs of children in Wales, piloting a measure of children’s health literacy within an existing nationwide health and well-being survey. This study aimed to examine and better understand the health literacy of primary school-aged children (aged 8–11 years) and explore associations between health literacy and health behaviours within this sample. The *HLSAC-5* was integrated within the *HAPPEN* survey and aims to provide a scalable and sustainable measure of children’s health literacy and health behaviours. Applying the *HLSAC-5* cut-points to *n* = 2607 children in this sample, 22.6% were categorized as having low health literacy, 50.4% moderate health literacy, and 27.0% high health literacy.

Findings suggest that compared to low health literacy, those with high health literacy were more likely to report more sleep, higher weekly physical activity, fewer sedentary days per week, and higher health-related well-being. Males were less likely to have high health literacy, while those in the least deprived quintile were more likely to have high health literacy. Moderate health literacy compared to low health literacy also had a statistically significant lower risk of being male, higher reported health-related well-being and brushing their teeth twice daily compared to zero times. The significance of this study is positioned within a research gap of assessment tools and understanding of the health literacy needs of pre-adolescent children. During this stage of middle childhood, children are gaining increasing independence and agency as they transition to adolescence. This is important because the knowledge, skills, and capacities crucial for optimal health literacy are developed during this formative developmental stage, and health behaviours are established that can be tracked into adolescence and adulthood that influence health and well-being throughout the life course (Bröder *et al*. 2017).

Almost three quarters of children in this sample (73%) had low or moderate health literacy, of which nearly one quarter (22.6%) were categorized as low. No comparable findings are currently available in Wales or the other UK nations for middle childhood, though wider European evidence within adolescent populations demonstrates significant cross-country variations. Using the *HLSAC-5*, data from seven countries ranged from low (9.0%–17.4%), moderate (53.8%–72.2%), and high health literacy (16.3%–37.3%) (Paakkari *et al*. 2020). While the findings in the current study are of concern given the higher proportion of those categorized as having low health literacy compared to international data, this may be due to the younger age of our sample. Though age was not a significant factor, developing health literacy occurs within a continuum of learning and development and is likely to increase with age (Bröder *et al*. 2017). Our findings should also be considered in the context of the validity of the *HLSAC-5* within this population. To date, the tool has been validated for use in ages 11 and above. The current study is the first to use the tool in children aged below 11 (8–11 years), and further validity research is required to examine its use during middle childhood. This represents the wider challenge of a lack of age-specific tools examining health literacy during middle childhood (Okan *et al*. 2018), limiting understanding of health literacy need across all stages of childhood. The current study aims to act as a catalyst for further research targeting this important developmental period influencing health literacy. Furthermore, variations in demographic characteristics between samples may exist. This includes the possibility of higher proportions of deprivation in the current study, given than deprivation rates in Wales are high, particularly in children with 28% living in relative poverty (Welsh Government 2024a, 2024b).

Regardless, low health literacy during childhood and adolescence is a key concern and is associated with a range of adverse health outcomes during childhood, adolescence, and throughout the life course. The majority of research to date examines this during adolescence, including lower self-reported health, fewer health-promoting behaviours, overweight and obesity and increased engagement in risk behaviours such as substance and alcohol use (Chang 2011, Fleary *et al*. 2018). Given the infancy of this research field, longitudinal research exploring associations between childhood and later adulthood health literacy is scarce. However, evidence suggests that cognitive, educational, and health markers during childhood are predictive of later adult health literacy (Solis-Trapala *et al*. 2023). Despite this, if low health literacy persists to adulthood, consequences include struggling to manage health information regarding disease prevention, health promotion, healthcare, and the management of long-term health conditions (Edwards *et al*. 2012, Baccolini *et al*. 2021). Low health literacy is associated with higher hospital admissions, duration of hospital stay, and likelihood of readmission, in addition to lower self-reported health status, poorer nutritional habits (i.e. fruit and vegetable consumption), and smoking status (Von Wagner *et al*. 2007, Shahid *et al*. 2022). As such, low health literacy during adulthood is a driver of higher healthcare costs. The findings of the current study should be considered in the context of the NHS in Wales, which is the Welsh Government’s largest area of expenditure (Stats Wales 2023).

While we advocate for greater health literacy, we do recognize that the environments people reside in can restrict health-promoting behaviours. Child health is driven not only by family and child health literacy, but also by aspects such as food availability [such as access to fast food (Jiang *et al*. 2023) or insecurity (Pool and Dooris 2022)], green space (Geary *et al*. 2023), housing conditions and stability (Gibson *et al*. 2011), and area air pollution (Milojevic *et al*. 2021). Hence, there is a possibility that contributors on a more macro-level restrict the ability for health literacy to play a role, and efforts to address population-level health literacy must extend beyond individual factors to that of place and policy. For *place-based* and *policy-focused* approaches in Wales, opportunities to develop children’s health literacy are present through ongoing major education system-level reforms and the rollout of the CfW (from September 2022 onwards). In particular, the new statutory focus on health and well-being as a curriculum area and overarching purpose provides a generational opportunity to enhance the health literacy of current and future generations. In the current study, health literacy survey items indicating levels of critical health literacy according to Nutbeam’s conceptualization (Nutbeam 2000) were reported as the lowest. The CfW framework strongly aligns with Nutbeam’s typology of health literacy; progression through the *Health and Well-being AoLE* continuum of learning requires children to develop from functional and interactive towards more critical health literacy domains (Welsh Government 2020, Marchant 2024). Given the school-level autonomy in curriculum design offered within the CfW, the findings from this study, in addition to ongoing knowledge exchange activities through *HAPPEN-Wales*, aim to facilitate schools in tailoring curriculum design to children’s health literacy and health behaviour needs, enabling schools to support learner progression through the development of functional, interactive, and critical health literacy (Nutbeam 2000). Addressing individual and context-specific health literacy needs is of significant importance as we move to a post-Covid ‘New Normal’ for education in Wales (Thomas *et al*. 2023).

From a health-promoting perspective, higher health literacy in our sample was associated with health-promoting behaviours including higher physical activity, sleep, and toothbrushing, and lower sedentary time. High levels of health literacy have been identified as one of the strongest explanatory variables for positive health outcomes (Fleary *et al*. 2018). This includes similar health indicators from the *HBSC* survey to those collected in this study, including physical activity, sleep and toothbrushing (Paakkari *et al*. 2019a). Although the study from Paakkari and colleagues was conducted in Finland and examined adolescent populations, it is important to note that this association remained significant when adjusted for structural factors including school achievement, educational aspiration, family affluence, age, and gender. The current study, which also accounted for school, area-level deprivation, age, and gender, adds to the evidence base by exploring middle childhood (aged 8–11 years). Further, children in our study residing in the least deprived quintiles had higher health literacy than those in the most deprived. This is unsurprising as a social gradient exists with health literacy unequally distributed across populations (Sørensen *et al*. 2015). It is well known within the wider health promotion literature that more favourable socio-economic conditions including access and availability to social and cultural resources support health-promoting behaviours and health status across the life course (Short and Mollborn 2015). Health literacy is also positioned among these as a social determinants of health (Coughlin *et al*. 2020), emphasizing the importance of addressing health literacy across the layers of socio-ecological influence (Rowlands *et al*. 2018, Dadaczynski *et al*. 2022). This includes individual (i.e. *person-centred*), organizational (i.e. *place-based*), and policy (i.e. *policy-focused*).

This study explored associations between health literacy and health behaviours, addressing an important gap as advocated for by the OECD, considering age-specific, child-reported measures that employ multiple indicators spanning health behaviours, perceptions, and well-being (OECD 2021).The health behaviours associated with higher health literacy in this study are all essential health-promoting behaviours required for optimal development and positive health and well-being outcomes during childhood and across the life course. For example, regular physical activity and lower sedentary behaviour during childhood are determinants of overweight and obesity (Chrissini and Panagiotakos 2021). These lifestyle-related predictors of cardiometabolic health are acquired during childhood (Deboer *et al*. 2015) and are risk factors for a range of non-communicable diseases (NCDs), responsible for the majority of chronic diseases and nearly three quarters of deaths worldwide (Robbins *et al*. 2021, WHO 2023b). In Wales, a quarter of children are overweight or obese in Wales (Public Health Wales NHS Trust 2023), and almost half of children (49%) do not meet physical activity guidelines (Richards *et al*. 2022). Sleep plays a crucial role in children’s physical, social, and behavioural health and is considered a fundamental requirement for healthy growth and development (Dahl 2007). Conversely, insufficient sleep during childhood is associated with a range of negative health implications including physical health, neurocognitive, emotional, and behavioural outcomes (Liu *et al*. 2024). Toothbrushing is an important behaviour associated with oral health outcomes, and evidence suggests that achieving the recommended frequency of twice-daily toothbrushing is an important preventative behaviour for tooth decay risk (Kumar *et al*. 2016, Baumgartner *et al*. 2022). Child oral health is a key public health concern in Wales, where over a third of children aged 5–6 years have tooth decay when starting primary school (Public Health Wales 2024). It is possible that the pathway between parental and child health literacy may play a role here (De Buhr and Tannen 2020, Marchant *et al*. 2022b). However, during middle childhood, children are gaining increasing independence and agency as they transition to adolescence. As such, the important role of adequate health literacy as a tool for developing the knowledge, skills, and competencies for engagement in health-promoting behaviours which develop during childhood should not be overlooked. Reducing the impact of low health literacy on the health service is essential and has potential in large healthcare savings. Targeting efforts earlier in childhood, such as through the CfW, may mitigate these future impacts by enhancing the health literacy of current and future generations. This is particularly pertinent given the widening of inequalities in children’s health, well-being, and education observed as a result of the COVID-19 pandemic, and the shorter- and longer-term impacts these may have on both children’s outcomes and the health service (James *et al*. 2021c, Marchant *et al*. 2021, 2022b).

Finally, children with higher health literacy in this study reported higher health-related well-being, measured using the Good Childhood Index by rating subjective happiness with health (The Children’s Society 2021). Indeed, both health and well-being are discussed synonymously among the literature as interwoven outcomes of optimizing health literacy (WHO Regional Office for Europe 2013). Further, well-being is embedded within the WHO’s definitions of both health and health promotion (WHO 1992). However, there appears a dearth of literature exploring associations between health literacy and well-being as a sole outcome, and even definitions and models of health literacy as a purpose of improving well-being are sparse despite well-being discussed in conjunction with health (Bröder *et al*. 2017). While conceptualizations of well-being are broad, findings from our study explored health-related well-being as a specific component. This could be interpreted as self-reported health status, and agrees with findings from Paakkari and colleagues, who, using the *HLSAC*, found that health literacy significantly explained self-rated health (Paakkari *et al*. 2019a).

### Strengths and limitations

This study is the first to explore health literacy among children in Wales and examine associations between health literacy and health behaviours. Findings from this study contribute to the evidence base by focusing on the understudied developmental period of middle childhood. During this period, the knowledge, skills, and capacities required for optimal health literacy are developed, and health behaviours are established that can be tracked into adolescence and adulthood, influencing health and well-being throughout the life course. However, a dearth of tools capable of assessing the health literacy of children and adolescents, and a significant lack of age-specific tools for middle childhood limit our understanding of children’s health literacy. The current study uses the five-item version of the *HLSAC*, developed by Paakkari *et al*. (Paakkari *et al*. 2016) in Finland, and translated, piloted, and validated in a number of European countries. The brevity of the *HLSAC-5* enables integration within existing population-level health-related surveys, as demonstrated within the *HAPPEN* survey in the current study. The conceptualization and domains of health literacy as a learning outcome represented within the *HLSAC-5* competencies strongly align with the new CfW framework, specifically regarding the *Health and Well-being AoLE*, providing a context-specific measure of children’s health literacy in Wales.

As noted in the *HLSAC* development paper and highlighted in wider reviews, many studies aimed at measuring health literacy in child and adolescent populations have not been validated in the specific target group (Perry 2014, Paakkari *et al*. 2016). Focus to date has been placed primarily on the adolescent period, with a dearth of tools exploring the health literacy needs during middle childhood (Okan *et al*. 2018). This challenge, highlighted by Okan and colleagues in a review of existing tools, requires urgent attention. Thus, our study aimed to contribute to this knowledge gap by integrating the *HLSAC-5* within the existing *HAPPEN* survey. At the time of writing, the *HLSAC* and *HLSAC-5* have been validated for use in populations aged 11 years and above (Bonde *et al*. 2022, Fischer *et al*. 2022, Masson *et al*. 2022). The current study extends understanding and applies the tool to children aged 8–11 years. It is important to note the *HLSAC-5* has not been validated in populations aged 10 and below. While school year and age were not significant factors in our model, *HLSAC-5* cut-points assigned for low, moderate, and high health literacy may vary within this age group. With this said, we had a Cronbach alpha of 0.88, suggesting high internal consistency, and our findings are consistent with wider *HLSAC-5* validation research in seven European countries, suggesting criterion validity in this context. The current study aims to act as a catalyst to further work targeting middle childhood, and this needs to be supported and informed by examining the *HLSAC-5* tool’s validity in younger childhood populations. This can strengthen knowledge in the field and ensure research captures this important developmental period contributing to health literacy.

Data from this study are cross-sectional using self-report methods and only represent the primary schools participating in this scoping and pilot study. Bias in self-reporting methods, the potential of social desirability in responses, and the representativeness of participating schools and the sample of children in our study must be acknowledged. Our sample may be biased towards more affluent groups with higher health literacy. Further longitudinal research is required across larger populations to further understand the health literacy of children and young people, and across the life course. *HAPPEN-Wales* continues to embed the *HLSAC-5* within its nationwide survey and thus, data collection is ongoing. Future longitudinal research extending cross-sectional *HAPPEN-Wales* data with cohort data and life course outcomes exploring factors that shape health literacy over the life course can be maximized, given the data linkage capabilities of *HAPPEN-Wales*, addressing an important research gap (Velardo and Drummond 2017). Developing a greater understanding of health literacy need at this age enables tailored practice and policy design and implementation. While this is particularly important in Wales with the prominence of health and well-being within a reformed curriculum, it also remains relevant to other contexts.

## CONCLUSION

This is the first study of its kind that captures and explores the health literacy needs of children in Wales. The integration of the *HLSAC-5* within the nationwide *HAPPEN* survey offers a scalable and sustainable measure of pre-adolescent children’s health literacy and health behaviours. Of the children in our study, 22.6% were categorized as having low health literacy, 50.4% as having moderate health literacy, and 27.0% as having high health literacy. Compared to those with low health literacy, those with higher literacy were more likely to report health-promoting behaviours including sleep, physical activity, toothbrushing, fewer sedentary days per week, higher health-related well-being, be female, and live in less deprived areas. The significance of this study is positioned within a gap of assessment tools and understanding of health literacy needs of pre-adolescent children (Bröder *et al*. 2017, Guo *et al*. 2018, Okan *et al*. 2018). This study contributes to the evidence base by focusing on the understudied developmental period of middle childhood and developing a greater understanding of health literacy need at this age. During this period, knowledge, skills, and capacities required for optimal health literacy are developed, and health behaviours are established that can be tracked into adolescence and adulthood that influence health and well-being throughout the life course (Bröder *et al*. 2017). The findings from this study and areas for future research exploring children’s health literacy needs should be considered within three key areas of attention: *person-centred* (i.e. understanding individual and population health literacy needs, specifically those of children and young people in Wales), *place-based* (i.e. examining specific organizational health literacy context within schools, including the role and impact of the CfW), and *policy-focused* (i.e. re-energizing health literacy in Wales by integrating health literacy within current and emerging policies and monitoring policy impacts).

In the short term, monitoring, tracking, and sharing school-level data of health literacy needs through integration of the *HLSAC-5* within the *HAPPEN* survey can support primary schools in tailoring health and well-being curriculum design and assessment, and place-based approaches to health literacy within the CfW. In the longer term, monitoring and tracking can improve understanding of population health literacy needs, ensuring that health literacy can be supported and enhanced at different developmental stages tailored to need, and inform the design and implementation of policy and practice. Combined with the implementation of the CfW alongside wider education system-level reforms, as well as wider public and population health ambitions, Wales thus offers a tractable and attractive national-scale health literacy policy testbed over the coming years (Marchant 2024).

## Supplementary Material

daaf026_suppl_Supplementary_File_A

daaf026_suppl_Supplementary_File_B

daaf026_suppl_Supplementary_File_C

## Data Availability

The data underlying this article will be shared on reasonable request to the corresponding author.
