# Supplementary material for: Examining the health literacy and health behaviours of children aged 8–11 in Wales, UK
Source: Health Promot Int. 2025 Apr 10;40(2):daaf026. doi: 10.1093/heapro/daaf026 (PMC11983690; doi:10.1093/heapro/daaf026)
Supplement: daaf026_suppl_Supplementary_File_A [file daaf026_suppl_supplementary_file_a.pdf]

The HAPPEN Survey

Consent Form
Before you start please click this link to read the information sheet...

https://happen-wales.co.uk/childrens-information-sheet/

1. I have read the child information sheet and understand that if I take part I can change my mind at any time, and this will not be a problem at all. *
Mark only one oval.

- Yes
- No

2. I am happy for you to use my questionnaire for research. Only the researchers in the team will know my name and will not tell anyone else my answers *
Mark only one oval.

- Yes
- No do not use my questionnaire

3. I am happy for you to look at my school and health records to see how my school is doing (as a group). This is anonymous which means I cannot be identified *
Mark only one oval.

- Yes
- No

If you do not wish to take part in the questionnaire please do not continue.

Please click next to start the questionnaire!

ABOUT YOU

4. First Name*

5. Last Name*

6. Home Post Code*

7. What school do you go to?*

8. Do you have any other children living in your house with you (brothers, sisters)?
Mark only one oval.

- Yes
- No

9. What year are you in now?*
Mark only one oval.

- Year 4
- Year 5
- Year 6

10. Do you have a garden?*

- Yes
- No

11. Gender*
Mark only one oval.

- Boy
- Girl
- Prefer not to say

12. Date of Birth
Year*
Mark only one oval.

- 2007
- 2008
- 2009
- 2010
- 2011
- 2012

13. Month*
Mark only one oval.

- January
- February
- March

- April
- May
- June
- July
- August
- September
- October
- November
- December

14. Day *
Mark only one oval.

- 1
- 2
- 3
- 4
- 5
- 6
- 7
- 8
- 9
- 10
- 11
- 12
- 13
- 14
- 15
- 16
- 17
- 18
- 19
- 20
- 21
- 22
- 23
- 24
- 25
- 26
- 27
- 28
- 29
- 30
- 31

YESTERDAY

15. How did you get to school yesterday?*
- On the bus
- On bike
- In the car/taxi

- Walked
- Ran/jogged
- Scooter
- Skateboarded/Rollerbladed

16. What did you have to eat for lunch yesterday?*

- School dinner
- Packed lunch from home
- Nothing

17. What did you do for most of your breaktimes yesterday?*

- Sat around inside or outside
- Ran around
- Stood around
- Walked around

18. How many friends did you play with yesterday?*

- I like to play on my own
- 1-2
- 3-4
- 5 or more

19. Did you have an afternoon break yesterday?*

- Yes
- No

20. How did you get home yesterday?*

- On the bus
- On bike
- In the car/taxi
- Walked
- Ran/jogged
- Scooter
- Skateboarded/Rollerbladed

AFTER SCHOOL

21. How may portions of fruit and vegetables did you eat yesterday?*

- 1
- 2
- 3
- 4
- 5

- 6
- 7
- 8

22. How many times did you brush your teeth yesterday?*

- 0
- 1
- 2
- 3

23. What time did you fall asleep YESTERDAY (to the nearest half hour)?
Mark only one oval.

- 6.00pm
- 6.30pm
- 7:00pm
- 7:30pm
- 8:00pm
- 8:30pm
- 9:00pm
- 9:30pm
- 10:00pm
- 10:30pm
- 11:00pm
- 11:30pm
- 12:00am
- 12:30am
- 1:00am
- 1:30am
- 2:00am
- 3:00am
- 3:30am
- 4:00am

24. What time did you wake up TODAY (to the nearest half hour)?
Mark only one oval.

- 5:00am
- 5:30am
- 6:00am
- 6:30am
- 7:00am
- 7:30am
- 8:00am
- 8:30am
- 9:00am
- 9.30am
- 10.00am
- 10.30am
- 11.00am

- 11.30am

THE LAST WEEK

25. In the last 7 days, how many days did you do sports or exercise for at least 1 hour in total. This includes doing any activities (this includes any activities or playing sports where your heart beat faster, you breathed faster, and you felt warmer?
Mark only one oval.

- 0 days
- 1-2 days
- 3-4 days
- 5-6 days
- 7 days

26. In the last 7 days, how many days did you watch TV/play online games/use the internet etc. for 2 or more hours a day (in total)?
Mark only one oval.

- 0 days
- 1-2 day
- 3-4 days
- 5-6 days
- 7 days

27. In the last 7 days, how many days did you feel tired?
Mark only one oval.

- 0 days
- 1-2 days
- 3-4 days
- 5-6 days
- 7 days

28. In the last 7 days, how many days did you feel like you could concentrate/pay attention well on your schoolwork?
Mark only one oval.

- 0 days
- 1-2 days
- 3-4 days
- 5-6 days
- 7 days
- Don't do school work

29. In the last 7 days, how many days did you drink at least one fizzy drink (e.g. coke, fanta, sprite)
Mark only one oval.

- 0 days
- 1-2 days
- 3-4 days

- 5-6 days
- 7 days

30. In the last 7 days, how many days did you eat at least one sugary snack (e.g. chocolate bar, sweets)
Mark only one oval.

- 0 days
- 1-2 days
- 3-4 days
- 5-6 days
- 7 days

31. In the last 7 days, how many days did you eat take away foods (e.g. Chinese takeaway)
Mark only one oval.

- 0 days
- 1-2 days
- 3-4 days
- 5-6 days
- 7 days

SPORT AND ACTIVITY

32. These questions are going to ask you how you feel about physical activity (This includes any activity where your heart beats faster, you breathe faster and you feel warmer)

- I want to take part in physical activity
- I feel confident to take part in lots of different physical activities
- I am good at lots of different physical activities
- I understand why taking part in physical activity is good for me

33. What motivates you the most to take part in physical activity and sport (ranked as Strongly Agree, Agree, Can't Say, Disagree, Strongly Disagree).

- I am driven by enjoyment
- I am driven by having fun with my friends
- I am driven by learning and improving my skills
- I am driven by competing against myself
- I am driven by playing and competing in a team

34. Is anyone else at home physically active/do they play a sport?
- Yes
- No

35. If yes, who is this?
- Mum
- Dad
- Brother
- Sister
- Aunty

- Uncle
- Grandparents

36. How many times do you take part in sports club outside of school a week?

- 0
- 1
- 2
- 3
- 4
- 5
- 6
- 7
- 8
- 9
- 10

37. Can you ride a bike without stabilisers?

- Yes
- No

38. Can you swim 25 metres without a float or armbands? (This is 1 length in a standard swimming pool)

- Yes
- No

YOU AND YOUR FEELINGS

39. Tell us if you agree or disagree with the following:

- I am doing well at school
- I feel part of my school community
- I have lots of choice over things that are important to me
- There are lots of things I'm good at
- I have someone to talk to at home
- I feel safe in school
- I have someone to talk to in school

40. On a scale of 0 to 10 (0 being very unhappy and 10 being very happy), how do you feel about
*Based on the Good Childhood Index by the Children's Society

41. Your Health
Mark only one oval.

- 0
- 1
- 2
- 3
- 4

- 5
- 6
- 7
- 8
- 9
- 10

42. Your School
Mark only one oval.

- 0
- 1
- 2
- 3
- 4
- 5
- 6
- 7
- 8
- 9
- 10

43. Your Family
Mark only one oval.

- 0
- 1
- 2
- 3
- 4
- 5
- 6
- 7
- 8
- 9
- 10

44. Your Friends
Mark only one oval.

- 0
- 1
- 2
- 3
- 4
- 5
- 6
- 7
- 8
- 9
- 10

45. Your Appearance
Mark only one oval.

- 0
- 1
- 2
- 3
- 4
- 5
- 6
- 7
- 8
- 9
- 10

46. Your Life
Mark only one oval.

- 0
- 1
- 2
- 3
- 4
- 5
- 6
- 7
- 8
- 9
- 10

47. These questions are going to ask you how you feel about your health:

I have good knowledge about my health:
- Not true at all
- Not quite true
- Somewhat true
- Absolutely true

I can compare health-related information from different sources:
- Not true at all
- Not quite true
- Somewhat true
- Absolutely true

I can judge how my own actions affect my surroundings (e.g. the natural environment):
- Not true at all
- Not quite true
- Somewhat true
- Absolutely true

When necessary I can find information about my health that is easy for me to understand:

- Not true at all
- Not quite true
- Somewhat true
- Absolutely true

I can give reasons for the choices I make regarding my health:

- Not true at all
- Not quite true
- Somewhat true
- Absolutely true

YOU AND YOUR FEELINGS

This part of the survey is going to ask you how you feel. There are no right or wrong answers. You should just pick the answer which is best for you.

Based on the Me and My Feelings Questionnaire (Deighton, Tymms, Vostanis, Belsky, Fonagy, Brown, Martin, Patalay, & Wolpert, 2012)

48. Remember, there are no right or wrong answers, just pick which is right for you.
Mark only one oval per row.

I feel lonely

- Never
- Sometimes
- Always

I cry a lot

- Never
- Sometimes
- Always

I am unhappy

- Never
- Sometimes
- Always

I feel nobody likes me

- Never
- Sometimes
- Always

I worry a lot

- Never
- Sometimes

- Always

I have problems sleeping

- Never
- Sometimes
- Always

I wake up in the night

- Never
- Sometimes
- Always

I am shy

- Never
- Sometimes
- Always

I feel scared

- Never
- Sometimes
- Always

I worry when I am at school

- Never
- Sometimes
- Always

I get very angry

- Never
- Sometimes
- Always

I lose my temper

- Never
- Sometimes
- Always

I hit out when I am angry

- Never
- Sometimes
- Always

I do things to hurt people

- Never
- Sometimes
- Always

I am calm

- Never
- Sometimes
- Always

I break things on purpose

- Never
- Sometimes
- Always

YOUR LOCAL AREA

49. On a scale of 0 to 10 (0 being not very safe and 10 being very safe), how safe do you feel playing in your area?
Mark only one oval.

- 0
- 1
- 2
- 3
- 4
- 5
- 6
- 7
- 8
- 9
- 10

50. From your house, can you easily walk to school?
Mark only one oval.

- Yes
- No

51. From your house, can you easily walk to a park (for example a field, grassy area)?
Mark only one oval.

- Yes
- No

52. From your house, can you easily walk to a leisure centre/sports centre?
Mark only one oval.

- Yes

- No

53. Can you play in all the places you would like to?

- I can play in all the places I would like to
- I can play in some of the places I would like to
- I can only play in a few places I would like to
- I can hardly play in any of the places I would like to

54. Are you happy with the area that you live in?

- Yes
- No

55. *Do you think the 20 mile per hour speed limit in Wales is a good thing?*

- *Yes*
- *No*
- *I don't know*

56. Why do you think this? (Open ended)

57. If you could change something to make you and your friends healthier and happier, what would you change... IN SCHOOL?

58. If you could change something to make you and your friends healthier and happier, what would you change... OUT OF SCHOOL?

Don't forget to press submit below!

We have some resources on our website if you would like to learn more or would like to speak to someone... https://happen-wales.co.uk/some-resources-for-you/ (https://happen-wales.co.uk/some-resources-for-you/)
