# Supplementary material for: Examining the health literacy and health behaviours of children aged 8–11 in Wales, UK
Source: Health Promot Int. 2025 Apr 10;40(2):daaf026. doi: 10.1093/heapro/daaf026 (PMC11983690; doi:10.1093/heapro/daaf026)
Supplement: daaf026_suppl_Supplementary_File_B [file daaf026_suppl_supplementary_file_b.docx]

## HLSAC-10 (current study)

- I have good knowledge about my health
- I am able to give examples of how to improve my health in my surroundings (e.g. a nearby place or area, family, friends)
- I can compare health-related information from different sources
- I can follow instructions given to me by healthcare people (e.g. nurses, doctors)
- I can give examples of things that promote good health
- I can judge how my own actions affect my surroundings (e.g. the natural environment)
- When necessary I can find information about my health that is easy for me to understand
- I can judge how what I do and the choices I make affects my health
- I know if information about health is right or wrong
- I can give reasons for the choices I make for my health

[Response options: Not true at all, Not quite true, Somewhat true, Absolutely true]

## HLSAC-10 (original)

- I have good information about my health
- When necessary, I am able to give ideas on how to improve health in my immediate surroundings (e.g. a nearby place of area, family, friends)
- I can compare health-related information from different sources
- I can follow the instructions given to me by healthcare personnel (e.g. nurse, doctor)
- I can easily give examples of things that promote health
- I can judge how my own actions affect the surrounding natural environment
- When necessary I find health-related information that is easy for me to understand
- I can judge how my behaviour affects my health
- I can usually figure out if some health information is right or wrong
- I can give reasons for choices I make regarding my health

[Response options: Not at all true, Not completely true, Somewhat true, Absolutely true]

## HLSAC-5 (current study)

- I have good knowledge about my health
- I can compare health-related information from different sources
- I can judge how my own actions affect my surroundings (e.g. the natural environment)
- When necessary I can find information about my health that is easy for me to understand
- I can give reasons for the choices I make regarding my health

[Response options: Not true at all, Not quite true, Somewhat true, Absolutely true]

## HLSAC-5 (original)

- I have good information about my health
- I can compare health-related information from different sources
- I can judge how my own actions affect the surrounding natural environment
- When necessary I find health-related information that is easy for me to understand
- I can give reasons for choices I make regarding my health

[Response options: Not true at all, Not quite true, Somewhat true, Absolutely true]
