# Supplementary material for: Examining the health literacy and health behaviours of children aged 8–11 in Wales, UK
Source: Health Promot Int. 2025 Apr 10;40(2):daaf026. doi: 10.1093/heapro/daaf026 (PMC11983690; doi:10.1093/heapro/daaf026)
Supplement: daaf026_suppl_Supplementary_File_C [file daaf026_suppl_supplementary_file_c.docx]

| **Reference: Low health literacy** | **Estimate** | **Std. error** | **p.** | **95% confidence intervals** | |
| --- | --- | --- | --- | --- | --- |
|  |  |  |  | **Lower bound** | **Upper bound** |
| **Moderate health literacy** | | | | | |
| ***School year*** | 1.14 | 0.10 | 0.20 | 0.94 | 1.38 |
| ***Male***  *Reference: Female* | 0.77 | 0.18 | 0.15 | 0.54 | 1.10 |
| ***Ethnicity***  *Black*  *White*  *Mixed*  *Reference: Asian* | 0.69  0.61  0.59 | 0.70  0.53  0.60 | 0.59  0.34  0.38 | 0.17  0.22  0.18 | 2.72  1.70  1.92 |
| ***WIMD quintile***  *2*  *3*  *4*  *5 (least deprived)*  *Reference: 1 (most deprived)* | 0.74  1.05  0.95  1.00 | 0.30  0.29  0.27  0.31 | 0.32  0.85  0.85  0.99 | 0.42  0.60  0.56  0.55 | 1.33  1.84  1.62  1.85 |
| ***Fruit and vegetable consumption*** | 0.97 | 0.05 | 0.58 | 0.89 | 1.07 |
| ***Daily toothbrushing frequency***  *1*  *2*  *≥3*  *Reference: 0* | 1.56  1.42  0.85 | 0.46  0.44  0.55 | 0.34  0.42  0.76 | 0.63  0.60  0.29 | 3.84  3.37  2.46 |
| ***Hours of sleep*** | 0.99 | 0.05 | 0.77 | 0.90 | 1.08 |
| ***Days physically active in previous 7 days***  *Reference: 0 days* | 0.95 | 0.08 | 0.54 | 0.82 | 1.11 |
| ***Days sedentary in previous 7 days***  *Reference: 0 days* | 1.00 | 0.07 | 0.99 | 0.87 | 1.15 |
| ***Days consumed carbonated drink in previous 7 days***  *Reference: 0 days* | 0.93 | 0.08 | 0.33 | 0.79 | 1.08 |
| ***Days consumed sugary snack in previous 7 days***  *Reference: 0 days* | 1.01 | 0.08 | 0.87 | 0.87 | 1.18 |
| ***Ride a bike***  *Reference: Can’t ride a bike* | 0.91 | 0.27 | 0.71 | 0.54 | 1.53 |
| ***Health-related wellbeing*** | 1.09 | 0.04 | 0.03 | 1.01 | 1.18 |
| **High health literacy** | | | | | |
| ***School year*** | 0.97 | 0.11 | 0.77 | 0.77 | 1.21 |
| ***Male***  *Reference: female* | 0.70 | 0.21 | 0.09 | 0.46 | 1.06 |
| ***Ethnicity***  *Black*  *White*  *Mixed*  *Reference: Asian* | 2.29  1.11  0.43 | 0.80  0.64  0.75 | 0.30  0.87  0.26 | 0.48  0.32  0.10 | 10.97  3.88  1.86 |
| ***WIMD quintile***  *2*  *3*  *4*  *5 (least deprived)*  *Reference: 1 (most deprived)* | 0.49  1.09  0.54  1.60 | 0.37  0.33  0.33  0.35 | 0.05  0.80  0.06  0.17 | 0.23  0.57  0.28  0.81 | 1.01  2.08  1.03  3.15 |
| ***Fruit and vegetable consumption*** | 1.05 | 0.05 | 0.34 | 0.95 | 1.17 |
| ***Daily toothbrushing frequency***  *1*  *2*  *≥3*  *Reference: 0* | 8.98  9.83  3.09 | 1.11  1.10  1.17 | 0.05  0.04  0.34 | 1.01  1.15  0.31 | 79.80  84.40  30.49 |
| ***Hours of sleep*** | 1.09 | 0.07 | 0.17 | 0.96 | 1.24 |
| ***Number of days physically active in previous 7 days***  *Reference: 0 days* | 1.14 | 0.09 | 0.15 | 0.95 | 1.36 |
| ***Number of days sedentary in previous 7 days***  *Reference: 0 days* | 0.94 | 0.09 | 0.47 | 0.80 | 1.11 |
| ***Number of days consumed carbonated drink in previous 7 days***  *Reference: 0 days* | 0.88 | 0.10 | 0.22 | 0.73 | 1.08 |
| ***Number of days consumed sugary snack in previous 7 days***  *Reference: 0 days* | 0.99 | 0.09 | 0.93 | 0.83 | 1.19 |
| ***Ride a bike*** | 1.20 | 0.34 | 0.60 | 0.62 | 2.31 |
| ***Health-related wellbeing*** | 1.53 | 0.06 | 0.00 | 1.36 | 1.72 |
